# *Capnocytophaga canimorsus* blebitis: case report and review of literature

**DOI:** 10.1186/s12886-021-01823-8

**Published:** 2021-01-26

**Authors:** Michael C. Yang, John Ling, Sameh Mosaed

**Affiliations:** Department of Ophthalmology, Gavin Herbert Eye Institute, University of California, Irvine, CA 92697 USA

**Keywords:** *Capnocytophaga canimorsus*, Blebitis, Trabeculectomy, Bleb perforation, Bleb-associated infections, Case report

## Abstract

**Background:**

*Capnocytophaga canimorsus* is a facultative anaerobic, slow-growing, capnophilic, Gram-negative bacillus, that is commonly found in the microflora of canine and feline oral cavities. *Capnocytophaga* infections are an emerging zoonotic disease that can cause fatal systemic infections in immunocompromised individuals. Localized ocular *Capnocytophaga* infections, including keratitis, blepharitis, and endophthalmitis, can lead to severe eye threatening situations. To our knowledge, there is currently no documented case of *Capnocytophaga canimorsus* blebitis with bleb perforation after trabeculectomy.

**Case presentation:**

Our case report and literature review features a novel case of *Capnocytophaga* blebitis that occurred after trabeculectomy, associated with close dog contact (i.e. face licking). The patient had underwent trabeculectomy 10 years prior and presented with conjunctival injection, perforated bleb, and hypotony. Overall, patient was medically treated subconjunctival vancomycin, gentamicin and moxifloxacin drops. Trabeculectomy revision was performed with good visual outcome. Bacterial cultures grew *Capnocytophaga canimorsus*.

**Conclusions:**

We discuss the strategies for diagnosis, treatment, and common risk factors for ocular *Capnocytophaga* infections. At-risk patients with ocular infections should be asked about close contact with dogs and cats; and treated promptly with the proper antibiotic regimen.

## Background

Pet ownership has many benefits, with some studies reporting a lower risk of cardiovascular disease and mortality with dog ownership [1]. This cohabitation also comes with its own risks; half of all Americans will be bitten at least once in their lifetime [2]. *Capnocytophaga canimorsus* is commonly found in canine oral microflora, and less commonly in cats. In certain studies, *C. canimorsus* could be cultured from oral secretions of approximately 26% of dogs and 18% of cats [3]. Recent reports have shown the prevalence to be up to 74% of dogs [4]. *C. canimorsus* can cause a wide variety of infections, with a majority being systemic infections and less than 10% being localized infections [5]. Many of these localized infections have been ocular; reports include blepharitis, keratitis, and endophthalmitis [6–8]. However, to our knowledge, there has not yet been a reported case of blebitis linked to *C. canimorsus*. We present a case of *C. canimorsus* blebitis with bleb perforation, which highlights the virulence of *C. canimorsus* and the need for pet owners with predisposing risk factors to be vigilant of ocular exposures.

## Case presentation

**Fig. 1:**
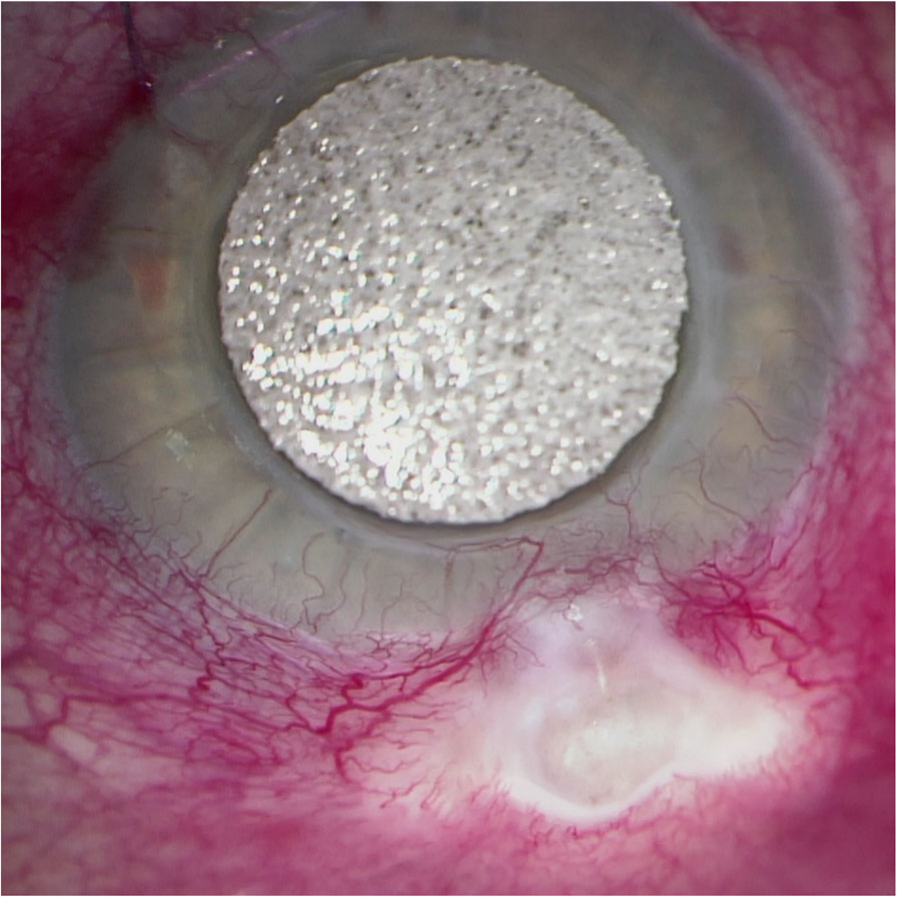
*Capnocytophaga* blebitis, preoperative clinical image. Significant injection, cystic bleb, with associated bleb leakage

An 81-year-old man was referred by an outside ophthalmologist with bacterial blebitis and perforated bleb, 10 years after trabeculectomy. Patient’s medical history was unremarkable. His ocular history includes radial keratotomy, penetrating keratoplasty (PKP), and the aforementioned trabeculectomy 10 years prior. Upon presentation, patient’s exam was notable for significant conjunctival injection, elevated cystic bleb, positive Seidel’s test with significant flow, and intraocular pressure of 4 mmHg (Fig. 1). Best corrected vision was 20/100. Corneal exam revealed fine keratic precipitates (KP) and evidence of PKP with mild haze around the periphery of the graft. Anterior chamber exam revealed trace cell and flare. Fundus exam was unremarkable except for cup-to-disc ratio of 0.85. No cells seen in vitreous. Given exam findings and concern for infection, patient was given subconjunctival injection of vancomycin 25 mg and gentamicin 20 mg. Conjunctival samples and scrapings were sent for fungal and bacterial cultures. Patient was prescribed a prolonged course of moxifloxacin drops; initially every 1 h and eventually tapered to four times a day. Prednisolone acetate drops were added, and the patient was monitored regularly in clinic with improvement to his clinical condition.

**Fig. 2:**
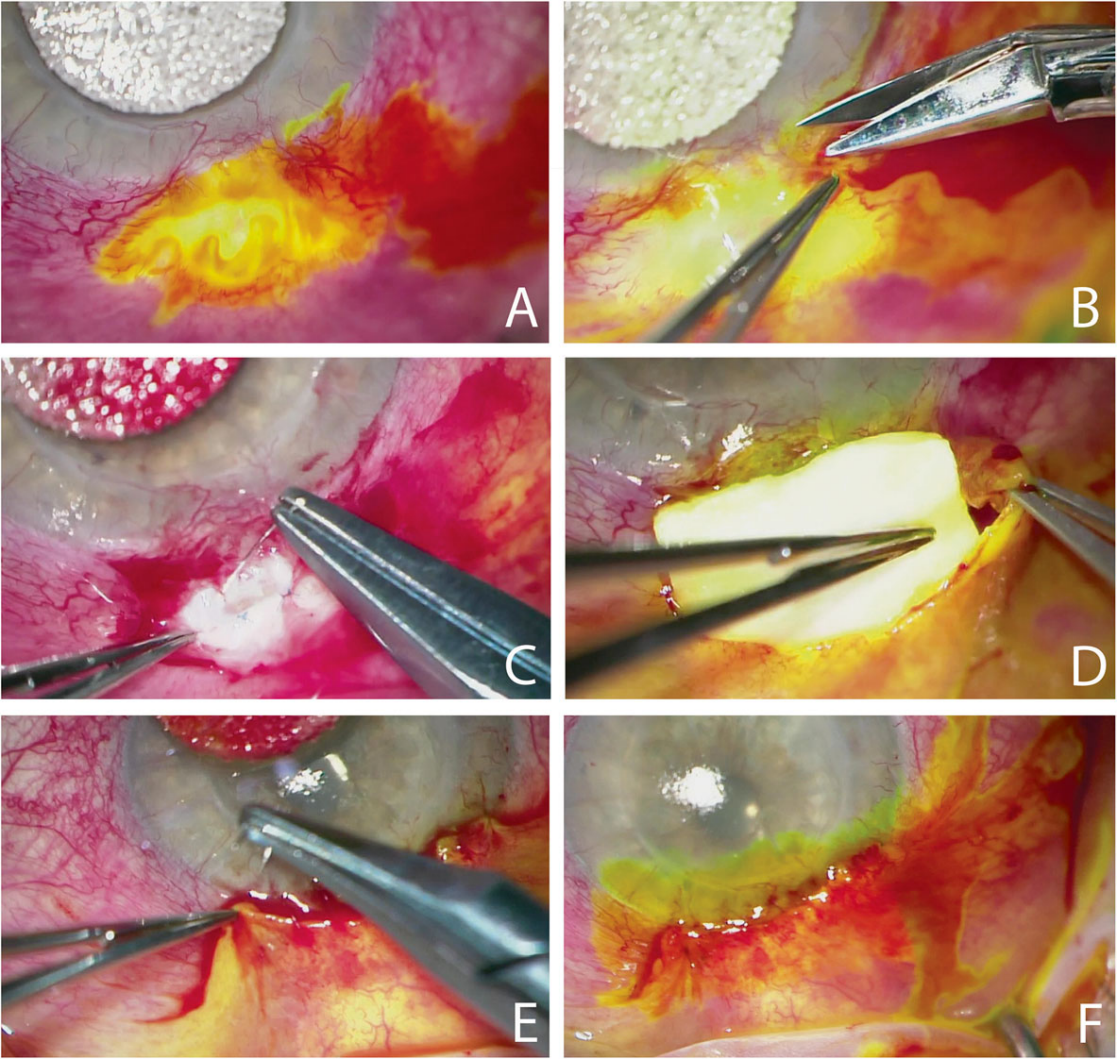
*Capnocytophaga* blebitis, intraoperative images of trabeculectomy revision. **a** Seidel positive, indicating bleb leakage. **b** Limbus dissection. **c** Flap closure with 10–0 Prolene suture, interrupted. **d** Placement of scleral patch graft secured with 8–0 Vicryl sutures. **e** Conjunctival closure with 10–0 Vicryl sutures, running. **f** Watertight closure confirmed with Seidel negative

**Fig. 3:**
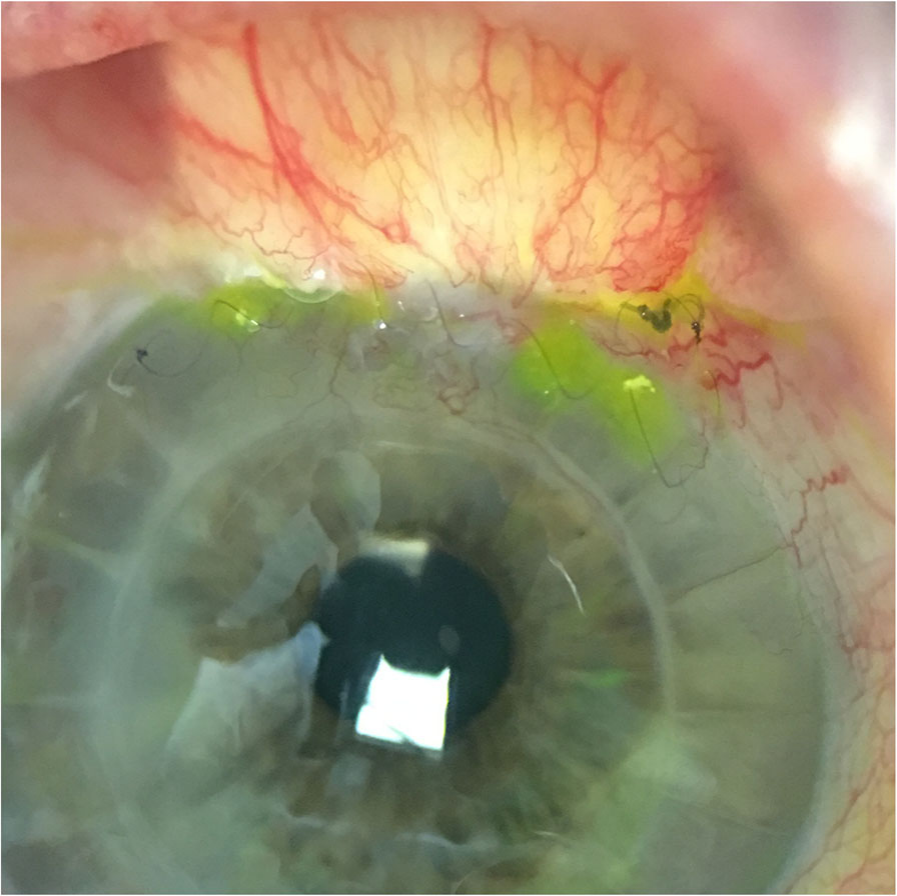
*Capnocytophaga* blebitis, 4 weeks postoperative clinical image. Status post bleb closure, excision of necrotic tissue and scleral patch graft. No bleb leakage, dissolving running suture visualized at limbus

Two weeks after initial presentation, trabeculectomy revision was performed involving closure of the bleb, excision of necrotic tissue and scleral patch graft (Fig. 2). Several months after surgery, patient continued to be Seidel’s negative with normal intraocular pressure, and best corrected vision was 20/70 (Fig. 3). Per standard protocol at our institution, samples were plated on sheep blood, chocolate, Mac Conkey agar, Brucella agar, phenyl ethyl alcohol agar, split plate (Bacteroides Bile Esculin, Laked Brucella agar with Kanamycin and Vancomycin), and thioglycolate. Given initial appearance as a rare bacillus species, cultures were sent to County of Orange, Health Care Agency, Public Health Laboratory for final identification of *C. canimorsus* species. Further details regarding identification and isolation were unavailable from the Public Health Laboratory — however, *C. canimorsus* has been known to grow on 5% sheep blood or chocolate agar in 5–10% CO_2_ at 37 C [5].

**Fig. 4:**
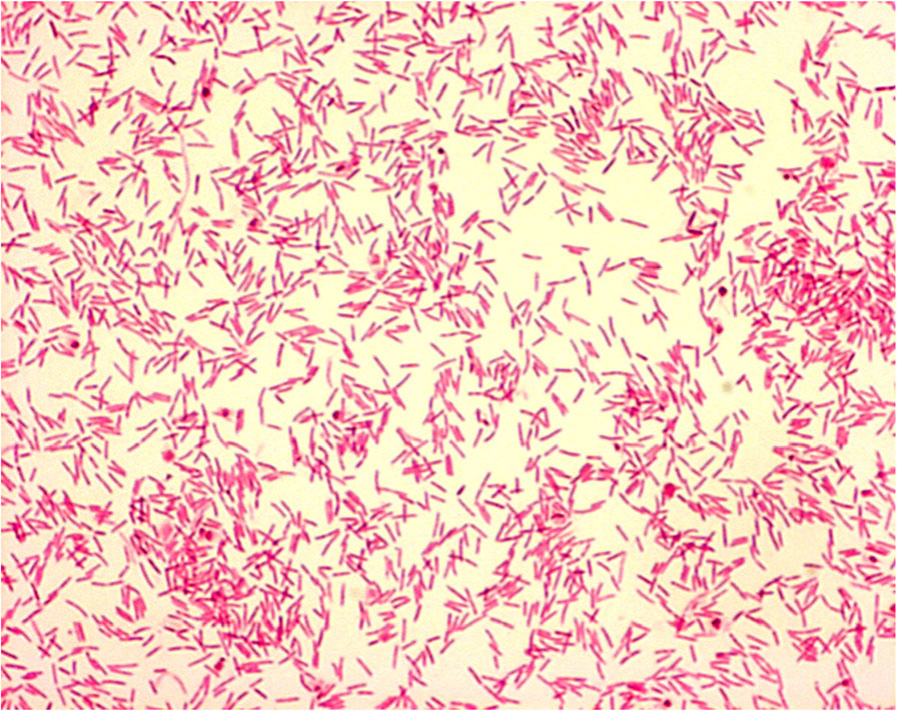
*Capnocytophaga canimorsus* Gram stain image. Gram negative rods, fusiform cells generally 1–3 μm in length. Sample culture image obtained with permission from *Microbe-Canvas* team

The diagnosis of *C. canimorsus* was initially suggested by appearance on Gram stain at our institution and final identification was made at aforementioned laboratory (Fig. 4). Further confirmation of *C. canimorsus* was obtained through DNA sequencing from an outside laboratory. The diagnosis of *Capnocytophaga canimorsus* was also confirmed independently by the referring ophthalmologist’s cultures and testing. Fungal cultures were negative. Upon further questioning, patient revealed that he had a Dalmatian dog with poor dentition that frequently licked his face.

## Discussion and conclusion

*Capnocytophaga canimorsus* is a Gram-negative bacillus that is capnophilic, a facultative anaerobe, and belongs in the *Bacteroidetes* phylum. It was originally named CDC group dysgonic fermenter 2 (DF-2), with articles published in the 1980s referring to *Capnocytophaga canimorsus* as DF-2. The first human infection was reported in 1976, and there has only been approximately 500 reported cases worldwide [9]. This is likely an underreported figure given that *C. canimorsus* is notoriously difficult to culture, and can also result in subclinical infections [10]. *C. canimorsus* can be transmitted through bites, scratches, and close animal contact (e.g. licking) [5].

With recent advances in ribosomal DNA sequencing, some researchers have proposed splitting *Capnocytophaga canimorsus* into two different species. The proposed name of the subclinical strain is *Capnocytophaga canis*, and maintaining the same name of *Capnocytophaga canimorsus* for the clinically symptomatic strain. However, recent case reports have isolated *C. canis* from patients with sepsis [11]. The strains that are harmful to humans all grow in heat-inactivated human serum, deglycosylate IgM and are cytochrome-oxidase positive [4]. Healthy, immunocompetent individuals are typically not susceptible to overt systemic *C. canimorsus* infections. However, at-risk individuals (e.g. immunocompromised, alcoholics, splenectomized patients) can have systemic *C. canimorsus* infections like endocarditis, meningitis, and even fatal bacteremia leading to multi-organ system failure [12–15].

Localized *C. canimorsus* infections have been mostly identified in the eye; keratitis being the most common presentation. *Capnocytophaga* keratitis is an aggressive cornea infection; in a study of ten patients, 50% required enucleation and 30% required corneal transplant [16]. Given its capnophilic nature, *Capnocytophaga* has a predilection for the deeper layers of the corneal stroma. Patients often present with corneal edema, endothelial lesions, ring-shaped infiltrates, and corneal perforation. Like our presented case, patients with *Capnocytophaga* keratitis report frequent and close interactions with dogs (e.g. face licking) [17]. Patients susceptible to *Capnocytophaga* keratitis are often immunocompromised—for example, status post bone marrow transplant, or rituximab infusions. Professions that are regularly exposed to canine and feline mouths (i.e. veterinarians) are also at risk [7]. Topical clindamycin has been used with good clinical results in some patients [7, 16, 17]. Some clinicians have also used oral clindamycin to achieve higher levels of antibiotics in the anterior chamber and deeper layers of the cornea where *Capnocytophaga* resides.

The reported incidence of bleb-associated infections (BAI) range from 1.5 to 4.8% at 5 years follow up. BAIs range from stage I with no cell in the anterior chamber and little to no visual impairment to stage IIIB with cell in the anterior chamber and vitreous with an obscured view of the fundus [18]. Early BAI occurs within the first post-operative month, and late BAI occurs after the first month. Our case of *Capnocytophaga* blebitis is a late BAI and stage II (cell in anterior chamber, no cell in vitreous). Diagnostic workup can include aqueous/vitreous stain and culture, polymerase chain reaction (PCR), biome representational in silico karyotyping (BRiSK), or metagenomic deep sequencing (MDS) [16–18]. The most commonly isolated microorganisms in early onset BAI is coagulase negative *Staphylococcus*, and late onset BAI is *Streptococcus.* There is currently no consensus on the most effective treatment for BAI. However, commonly used treatment regimens are fortified aminoglycosides (e.g. tobramycin (14 mg/mL) with vancomycin (q30 min) or 4th generation fluoroquinolone (q1 hour) [19]. These antibiotics broadly cover Gram-negative and Gram-positive microorganisms. Of note, *Capnocytophaga’s* susceptibility to aminoglycosides and vancomycin varies widely between studies; but is typically susceptible to clindamycin, penicillin, cephalosporin, imipenem, and beta-lactamase inhibitor combinations [7, 20]. Our patient was initially treated with subconjunctival vancomycin and gentamicin; and eventually shifted to a prolonged course of topical moxifloxacin with treatment success. Subconjunctival antibiotics are not routinely administered; however, our patient was elderly and the typical regimen of q30 min eyedrops of vancomycin and aminoglycoside was not possible. Additionally, given the urgency of acute blebitis and the importance of prompt administration of antibiotics, we opted for a broad spectrum approach with vancomycin and gentamicin before culture sensitivities were available. Given its high lipophilicity, topical moxifloxacin achieves high intraocular concentrations in aqueous humor and conjunctiva. Moxifloxacin covers broadly for Gram-negative, Gram-positive and atypical pathogens [18].

*Capnocytophaga canimorsus* is a member of the complex microflora of canine and feline oral cavities. In immunocompromised patients, *C. canimorsus* has been known to cause severe systemic infections like bacteremia and multi-organ failure. In rare cases, *C. canimorsus* can cause eye-threatening ocular infections ranging from keratitis to endophthalmitis. Our novel case of *Capnocytophaga* blebitis contributes to the scarce clinical information available regarding ocular *Capnocytophaga* infections. At-risk patients with ocular infections should be asked about close contact with dogs and cats; and treated promptly with the proper antibiotic regimen.

## Data Availability

Data sharing is not applicable to this article as no datasets were generated or analyzed during the current study.

## Abbreviations

PKP: Penetrating keratoplasty
KPP: Keratic precipitates
CDCP: Centers for Disease Control
DF-2P: Dysgonic fermenter 2
BAIP: Bleb-associated infections
PCRP: Polymerase chain reaction
BRiSKP: Biome representational in silico karyotyping
MDSP: Metagenomic deep sequencing
